# Sedentary Thresholds for Accelerometry-Based Mean Amplitude Deviation and Electromyography Amplitude in 7–11 Years Old Children

**DOI:** 10.3389/fphys.2019.00997

**Published:** 2019-08-07

**Authors:** Ying Gao, Eero A. Haapala, Anssi Vanhala, Arja Sääkslahti, Merja Rantakokko, Arto Laukkanen, Arto J. Pesola, Timo Rantalainen, Taija Finni

**Affiliations:** ^1^Faculty of Sport and Health Sciences, University of Jyväskylä, Jyväskylä, Finland; ^2^Institute of Biomedicine, University of Eastern Finland, Kuopio, Finland; ^3^School of Health and Social Studies, JAMK University of Applied Sciences, Jyväskylä, Finland; ^4^Active Life Lab, South-Eastern Finland University of Applied Sciences, Mikkeli, Finland

**Keywords:** resting energy expenditure, accelerometry, electromyography, sitting, standing, posture

## Abstract

We investigated the ability of energy expenditure, movement sensing, and muscle activity to discriminate sedentary and non-sedentary activities in children. Thirty-five 7–11-year-old children participated in the study. Simultaneous assessment of oxygen uptake (V̇O_2_), triaxial accelerometry, and thigh muscle electromyography (EMG) were performed during eight different sedentary and non-sedentary activities including lying down, sitting-, standing-, and walking-related activities, which were performed in a random order. Mean values of V̇O_2_, accelerometry, and EMG from the concurrent 2 min epochs during each activity were computed. Resting energy expenditure (REE) was measured during 30 min supine rest. Directly measured metabolic equivalent of tasks (METs, V̇O_2_ in activities/V̇O_2_ in REE) were calculated for each activity. Mean amplitude deviation (MAD) was computed for accelerometry. EMG was normalized for mean muscle activity during self-paced walking. The classification accuracy of METs, MAD, and EMG to discriminate sedentary activities from physical activities was investigated by receiver operating characteristic curves and optimal cut-offs based on maximal sensitivity and specificity. Mean (SD) REE was 5.0 ± 0.8 ml/kg/min. MET, MAD, and EMG values ranged from 1.0 to 4.9, 0.0020 to 0.4146 g, and 4.3 to 133.9% during lying down and walking at 6 km/h, respectively. Optimal cut-offs to discriminate sedentary activities from non-sedentary activities were 1.3 for METs (sensitivity = 82%, specificity = 88%), 0.0033 g for MAD (sensitivity = 80%, specificity = 91%), and 11.9% for EMG (sensitivity = 79%, specificity = 92%). In conclusion, this study provides applicable thresholds to differentiate sitting and standing and sedentary and non-sedentary activities based on METs, MAD, and EMG in young children.

## Introduction

Sedentary lifestyle has reached pandemic levels among children across the world (Carson et al., 2016). The evidence from studies using accelerometry to assess sedentary behavior consistently suggests that children and adolescents spend most of their waking hours being sedentary (Cooper et al., 2015; LeBlanc et al., 2015). However, the prevalence of sedentary behavior and the magnitude of the associations between sedentary behavior and health outcomes are modified by the utilized accelerometer cut-offs (Atkin et al., 2013; Banda et al., 2016). Therefore, accurate assessment and definition of sedentary behavior are necessary in the studies on the associations of sedentary behavior with different health outcomes and when creating sedentary behavior and physical activity surveillance systems in children (Salmon et al., 2011).

Sedentary behavior is defined as any waking behavior in a sitting, reclining, or lying posture with energy expenditure less than 1.5 metabolic equivalents of task (MET; Tremblay et al., 2017). One MET is usually considered as equal to an oxygen uptake (V̇O_2_, ml/kg/min) during peaceful sitting or lying down (Jetté et al., 1990). MET of 1.5 has been found to relatively accurately discriminate sitting from standing in adults (Mansoubi et al., 2015). Furthermore, some previous studies suggest that energy expenditure alone is not accurate in assessment of sedentary activities and including postures would enhance the discrimination accuracy (Pesola et al., 2016; Gao et al., 2017).

Because free-living measurement of V̇O_2_ is not feasible, accelerometry has become the most common method to assess sedentary behavior (Migueles et al., 2017). The mean amplitude deviation (MAD) method is used to compare data gathered by different types of accelerometers because it utilizes universal *g* values instead of arbitrary counts (Vähä-Ypyä et al., 2015b). To the best of our knowledge, few studies have studied the validity of MAD in classification of sedentary and physical activities (Aittasalo et al., 2015; Vähä-Ypyä et al., 2015a), and none of them had utilized V̇O_2_ to cross-validate MAD values against V̇O_2_ in children. Furthermore, instead of measuring physiological parameters such as energy expenditure and muscle activity or inactivity, accelerometry only captures movement (Godfrey et al., 2008). That is, it is well established that changing from a lying or sitting posture to a standing posture increases energy consumption by about a 50% due to muscles having to overcome the pull of gravity. Standing is a stationary activity, which therefore does not register on an accelerometer, and this increased in energy expenditure is not reflected in the accelerometer readings. Low energy expenditure and muscle inactivity are the underlying mechanisms in the relationships between high levels of sedentary behavior and impaired health (Hamilton et al., 2007; Hamilton, 2017).

Measuring muscle activity using electromyography (EMG) may provide more direct information on sedentary behavior and physical activity than accelerometry (Hamilton et al., 2007; Hamilton, 2017). We have previously found that EMG may provide superior accuracy in the assessment of low intensity physical activity and to better capture typical short-lasting sporadic activity bouts than accelerometry in children (Gao et al., 2018). Furthermore, previous studies from our laboratory have determined EMG thresholds for sedentary activities using data derived from adults (Tikkanen et al., 2013, 2014; Pesola et al., 2016), but such thresholds have not been developed for children.

Our understanding of sedentary behavior of children is still limited because of the lack of comprehensive studies with concurrent assessment of energy expenditure, accelerometry, and muscle activity. The primary aim of the present study was to establish the optimal cut-offs for sedentary activities in children using energy expenditure, accelerometry, and EMG. We therefore investigated the ability and accuracy of energy expenditure defined as METs, accelerometry-derived MAD, and thigh muscle activity to discriminate sedentary and non-sedentary activities in children. We hypothesized that (1) MAD can be used to differentiate sitting and standing, and sedentary and non-sedentary activities in children (Mansoubi et al., 2015) but (2) energy expenditure and muscle activity will be more sensitive to discriminate different sedentary activities from each other and sedentary activities from non-sedentary activities than accelerometry (Pesola et al., 2016).

## Materials and Methods

### Participants

This study is a part of the Children’s Physical Activity Spectrum (CHIPASE) study. Children were recruited from local schools. Forty-five children and their families were interviewed in the familiarization session, and 10 of them withdrew due to scheduling difficulties. Finally, 35 healthy children aged 7–11 years who volunteered to participate and were included in the study. All aspects of the CHIPASE study were approved by the Ethics Committee of the University of Jyväskylä. All children gave their assents, and their parents/caregivers gave their written informed consents. The study was conducted in agreement with the Declaration of Helsinki.

### Power Calculations

A sample size of 30 was estimated to provide sufficient statistical power for differentiating METs between sitting (1.33 ± 0.24) and standing (1.59 ± 0.37) based on the data of Mansoubi et al. (2015) with 80% power and 5% *α-*error level.

### Overview of the Protocol

The participants visited laboratory for familiarization session and for two measurement sessions.

#### Familiarization Session

The participants and their parents were introduced to the study protocol and got familiarized to the laboratory environment and measurement devices. They also provided written informed consent during the visit.

#### Measurement Visit 1

The participants arrived at the laboratory in the morning after 10–12 h overnight fast. Stature was measured to the nearest 0.1 cm using a stadiometer. Body mass, skeletal muscle mass, fat mass, fat free mass, and percent body fat were measured with a bioelectrical impedance device (InBody 770, Biospace Ltd., Seoul, Korea). Body mass index standard deviation score (BMI-SDS) was computed using the Finnish reference values (Saari et al., 2011). After these assessments, participants were helped to dress in EMG shorts (Myontec Ltd., Kuopio, Finland), and an elastic belt with an accelerometer (X6-1a, Gulf Coast Data Concepts Inc., Waveland, USA) worn on the right hip. Resting energy expenditure (REE) was measured over 30 min when children were lying down in a supine position in a quiet room with a stable temperature. Children were allowed to watch a children’s program from a digital device, and the program was the same for all children. Respiratory gases were collected using a pediatric face mask (Hans Rudolph, Inc., Kansas, USA) and recorded using a respiratory gas analyzer (Oxycon mobile, CareFusion Corp, USA). After the assessment of REE, a breakfast was served for children. The validation against Douglas Bag method has shown that Oxycon mobile is reliable and valid in respiratory gas exchange analysis (Rosdahl et al., 2010).

#### Measurement Visit 2

At the second visit, the arrival time was not standardized, and the participants arrived at the laboratory when it suited to their schedule. Children were asked to perform the following activities for 4.5 min in a random order interspersed with 1-min rest (Saint-Maurice et al., 2016): sitting quietly, sitting while playing a mobile game, standing quietly, standing while playing a mobile game, walking on a treadmill at 4 and 6 km/h, and self-paced walking around an indoor track (on an average of 5.0 ± 0.8 km/h). V̇O_2_, MAD, and EMG were concurrently recorded during the tasks.

### Measurement of Oxygen Uptake, Accelerometry, and Electromyography

All activities were timed and recorded in a log sheet. Devices were synchronized using a custom-written Matlab (MathWorks, MA, USA) script based on the recording sheets. Synchronization was confirmed visually and re-synchronized manually if necessary. The raw data of V̇O_2_, MAD, and EMG were averaged into non-overlapping 1 s epochs for each activity prior to calculating the 2-min mean values that were used as the outcome measures.

#### Indirect Calorimetry

The respiratory gas analyzer was calibrated according to manufacturer’s guidelines before assessments. Dead space was adjusted to 78 ml for the petite size of the face mask following the manufacturer’s recommendations. V̇O_2_ (ml/kg/min), carbon dioxide production (V̇CO_2_, ml/kg/min), and respiratory exchange ratio (RER) were collected breath by breath and computed in non-overlapping 1 s epoch length. Data collected during third and fourth minute when plateau in V̇O_2_ and V̇CO_2_ was observed. V̇O_2_ was then averaged over 2 min and used for analyses (Saint-Maurice et al., 2016). V̇O_2_ in different activities was converted to MET values. Those values were calculated based on individual REE measured METs (V̇O_2_ measured during the activities/V̇O_2_ in REE). REE was determined for the mean value between the 15th and 25th minute of 30 min laying down when the steady state was reached (Ventham and Reilly, 1999). Otherwise, the steady state was visually selected for further analysis.

#### Triaxial Accelerometry

The triaxial accelerometry was provided as the raw acceleration data in actual g units, where the high range up to 6 g with 16-bit A/D conversion and sampling at 40 Hz. The resultant acceleration of the triaxial accelerometer signal was calculated from $\sqrt{x^{2}+y^{2}+z^{2}}$, where *x*, *y*, and *z* are the measurement sample of the raw acceleration signal in *x*, *y*, and *z* directions. The number of consecutive data points was 40, and corresponding epoch length was 1 s. The X6-1a accelerometer has been confirmed concurrent validity with ActiGraph GT3X accelerometer in children (Laukkanen et al., 2014). The universal analysis of MAD was calculated from the resultant acceleration in non-overlapping 1 s epoch. MAD described as the mean distance of data points about the mean ($\frac{1}{n}\sum_{i=1}^{n}\left|r_{i-}\bar{r}\right|$, where *n* is the number of samples in the epoch, *r_i_* is the *i*th resultant sample within the epoch, and $\bar{r}$ is the mean resultant value of the epoch; Aittasalo et al., 2015; Vähä-Ypyä et al., 2015b). Thus, the mean of MAD values (g) was calculated in the certain 2-min time window for each activity and 10 min for lying down.

#### Textile Electromyography

Textile EMG electrodes embedded into elastic garments were used to assess muscle activity from the quadriceps and the hamstring muscles. Four different sizes of EMG shorts (120, 130, 140, and 150 cm) with using zippers located at the inner sides of short legs and adhesive elastic band in the hem ensured proper fit in every child. The conductive area of the electrodes over the muscle bellies of the left and the right quadriceps was 9 × 2 cm^2^ (length × width) in all short sizes, while the corresponding sizes for the hamstring muscles were 6 × 2 cm^2^ in sizes of 120, 130, and 140 cm and 6.5 × 2 cm^2^ in size of 150 cm. The conductive area of the reference electrodes was 11 × 2 cm^2^, and they were located longitudinally over the iliotibial band. Water or electrode gel (Parker Laboratories Inc., Fairfield, NJ, USA) was used on the electrode surfaces to minimize the skin-electrode impedance.

EMG signal was stored in a small waist-mounted module (Finni et al., 2007) and sampled at 1,000 Hz after which the data were pre-processed into non-overlapping 40 ms root-mean-squared values. This technology has been reported to be valid, reproducible, and feasible in adults (Finni et al., 2007; Pesola et al., 2014) and to have good day-to-day reliability in children (Gao et al., 2018). Data were downloaded to Muscle Monitor software provided by the manufacturer (Myontec Ltd., Kuopio, Finland) and visually checked for possible artifacts and non-physiologic signals. If the artifacts lasted more than the analyzed duration in a specific activity, then it was manually discarded from the particular channel. Baseline shifts were corrected based on a moving 5-min window (Tikkanen et al., 2013). The 5-min window was determined to be the best to correct for minor baseline fluctuations without distorting the physiological signal (Pesola et al., 2014). In the signal analysis, EMG data were identified from different activities in the certain time windows simultaneously according to the steady state in respiratory gases. Individual EMG activities were normalized channel by channel to EMG amplitude measured during self-paced walking (%EMG_self-paced walking_). The normalized EMG data were averaged for quadriceps from right and left side and hamstring muscles from right and left side, then the mean amplitude of the average normalized data was computed as the intensity of muscle activity level for each activity.

### Statistics

Statistical analyses were conducted using IBM SPSS for Windows 24.0 (IBM Corp., Armonk, NY, USA). The data were described as mean ± standard deviation (SD) or mean with 95% confidence interval (CI) unless otherwise indicated. Normality of the data was investigated with Shapiro-Wilk test. Independent samples *t* test was used to compare sex differences. METs, MAD, and EMG were normalized for corresponding measure during self-paced walking to allow comparison between methods.

Two-way repeated measures analysis of variance (ANOVA) was used to compare differences between the measures of METs, MAD, and EMG within specific activities including lying down vs. standing quietly, sitting quietly vs. standing quietly, and during sitting or standing quietly vs. while playing mobile game. When ANOVA revealed significant main effects, *post hoc* comparisons by a Bonferroni correction were used to localize the difference. A probability level of *p* ≤ 0.05 (two-tailed) was considered statistically significant.

Receiver operating characteristics (ROC) curves were used to investigate the optimal cut-offs for METs, MAD, and EMG to discriminate sedentary activities from non-sedentary activities. Sedentary activities were pre-determined based on measured energy expenditure (≤1.5 METs) and non-upright postures. We also performed ROC curves analyses excluding walking-related activities from non-sedentary activities to discriminate lying down or sitting from standing-related activities. The area under the curve (AUC) with their 95% CI is considered a measure of the utility of the predictor variable and represents the trade-off between the correct identification of sedentary activity (sensitivity) and the correct identification of non-sedentary activity (specificity). The cut-off that maximized the norm of sensitivity and specificity (that is, the cut-off that resulted in the maximum value of the square root of the sum of the sensitivity squared and specificity squared) is reported. An AUC of 1 represents the ability to perfectly identify sedentary activities from non-sedentary activities, whereas an AUC of 0.5 indicates no greater predictive ability than chance alone (Fan et al., 2006).

Spearman’s rho (*r*) was individually determined for all tasks and activities between METs and MAD, METs, and EMG. Mean correlation coefficient was averaged from individual correlation coefficients. The strength of correlation was interpreted as weak (<0.30), low (0.30–0.49), moderate (0.50–0.69), strong (0.70–0.89), or very strong (>0.90) (Pett, 1997).

#### Missing Values

Data were initially screened for missing values for each activity. In one case, we observed an abnormal REE value, which was then predicted from others based on age, sex, height, body mass, and fat free mass. Of the 280 activities (35 participants × 8 activities), acceptable data were obtained for a total of 242 activities. Full datasets of concurrently recorded both measured and adults METs, MAD and EMG were obtained for 84 pre-determined sedentary activities and 158 non-sedentary activities.

## Results

Boys were heavier (*p* = 0.009) and had more skeletal muscle mass (*p* = 0.009) and more fat-free mass (*p* = 0.012) than girls (Table 1). There were no other differences between boys and girls.

**Table 1:** Characteristics of participants.

Mean ± SD	All (*n* = 35)	Girl (*n* = 21)	Boy (*n* = 14)
Age (years)	9.6 ± 1.4	9.6 ± 1.5	9.7 ± 1.4
Stature (cm)	137.6 ± 9.2	135.7 ± 9.3	140.4 ± 8.7
Body mass (kg)	32.6 ± 6.9	30.2 ± 6.0	36.2 ± 6.8^†^
Skeletal muscle mass (kg)	14.0 ± 2.9	13.0 ± 2.5	15.5 ± 2.8^†^
Body fat mass (kg)	5.7 ± 3.6	4.9 ± 3.0	6.8 ± 4.2
Fat free mass (kg)	26.9 ± 4.8	25.2 ± 4.2	29.4 ± 4.6^†^
BMI standard deviation score^*^	−0.2 ± 1.2	−0.5 ± 1.1	0.3 ± 1.2
Percent body fat (%)	16.6 ± 8.1	15.7 ± 7.3	18.0 ± 9.3
RER during REE	0.883 ± 0.124	0.884 ± 0.145	0.882 ± 0.089
V̇O_2 REE_ (ml/kg/min)^#^	5.0 ± 0.8	4.9 ± 0.6	5.1 ± 1.1

* *BMI standard deviation score was calculated based on Finnish age and sex specific growth charts (Saari et al., 2011)*.

# *One case of abnormal resting energy expenditure (REE) value was predicted from others based on age, gender, height, body mass, and fat free mass*.

† *Significant difference between genders, p < 0.05*.

### Metabolic Equivalent of Tasks, Mean Amplitude Deviation, and Electromyography During Sedentary and Non-sedentary Activities

**Figure 1:**
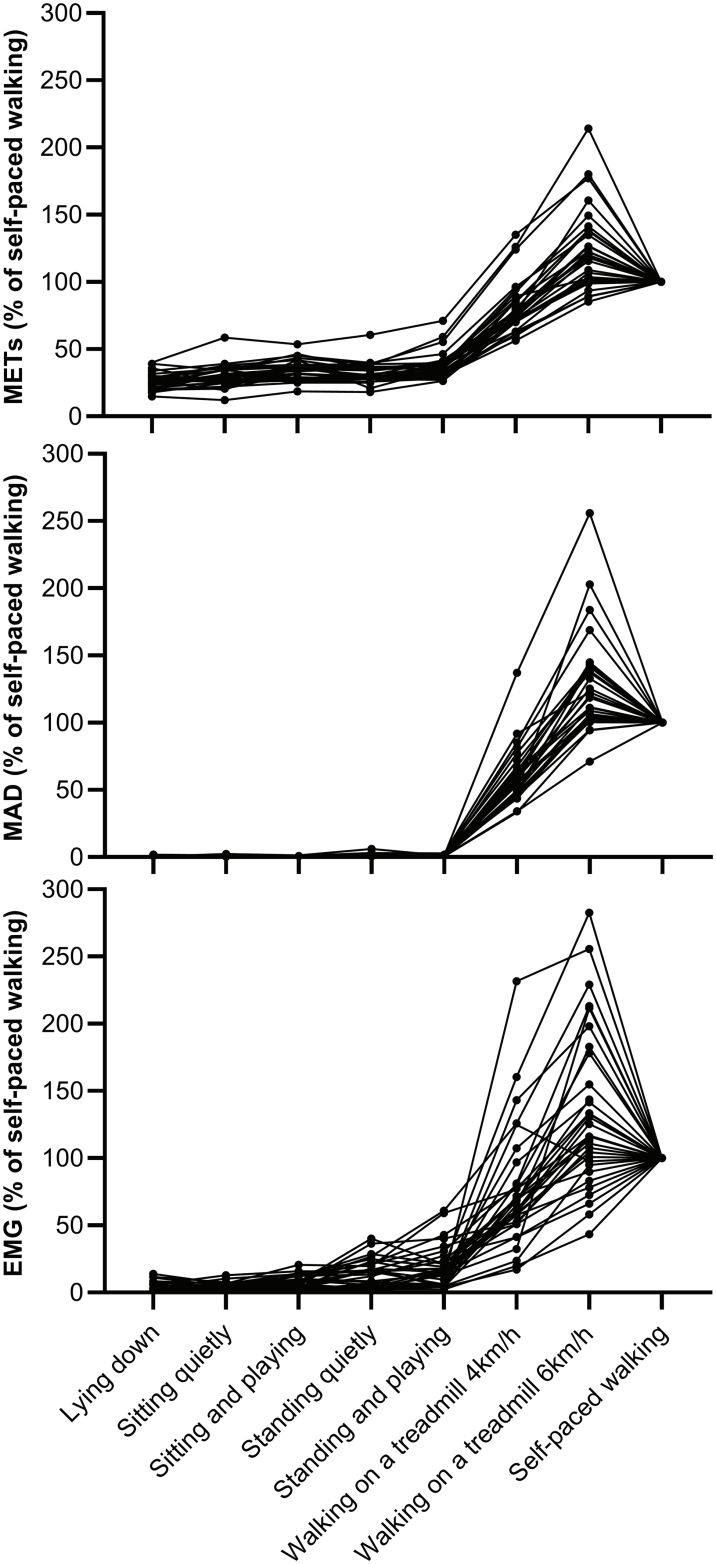
Individual values of METs, MAD, and EMG during different activities normalized for corresponding measure during self-paced walking. Each plot and line correspond to an individual child.

The mean (SD) of REE in children was 5.0 ± 0.8 ml/kg/min. The results of METs, MAD (g), and EMG (%) for each activity are presented in Table 2. When we compared METs, MAD, or EMG between lying down and sitting- and standing-related activities, we found significant main effects (all *p* < 0.001) for METs, MAD, and EMG in all activities (Figure 1). METs, MAD, and EMG were lower during lying down and sitting quietly than during standing quietly (both *p* < 0.05). METs and EMG were also lower during sitting quietly than sitting while playing a mobile game (both *p* < 0.001) and during standing quietly than during standing while playing a mobile game (both *p* ≤ 0.05). There were no statistically significant differences in MAD between either sitting quietly and sitting while playing a mobile game or between standing quietly and standing while playing a mobile game (both *p* > 0.05).

**Table 2:** The directly measured metabolic equivalent of tasks (METs), mean amplitude deviation (MAD), and mean muscle activity (EMG) in different sedentary and non-sedentary activities.

All activities (mean ± SD)	METs	MAD (g)	EMG (%)
Lying down (REE; *n* = 35/34/34)	1.0 ± 0.0	0.0020 ± 0.0011	4.3 ± 3.6
Sitting quietly (*n* = 34/32/32)	1.2 ± 0.2	0.0021 ± 0.0012	4.3 ± 2.8
Sitting while playing mobile game (*n* = 34/33/32)	1.3 ± 0.2	0.0024 ± 0.0009	7.4 ± 5.1
Standing quietly (*n* = 33/33/32)	1.3 ± 0.2	0.0046 ± 0.0033	14.1 ± 10.1
Standing while playing mobile game (*n* = 34/33/32)	1.5 ± 0.3	0.0041 ± 0.0022	18.3 ± 15.3
Walking on a treadmill at 4 km/h (*n* = 33/33/32)	3.2 ± 0.7	0.1932 ± 0.0363	75.2 ± 43.9
Walking on a treadmill at 6 km/h (*n* = 34/33/32)	4.9 ± 1.0	0.4146 ± 0.0718	133.9 ± 58.1
Self-paced walking^*^ (*n* = 31/31/30)	4.1 ± 1.0	0.3353 ± 0.0705	100.0 ± 0.0

* *Self-paced walking around an indoor track, individual speed was an average of 5.0 ± 0.8 km/h*.

### Optimal Cut-Offs for Sedentary Thresholds in Different Measures

**Figure 2:**
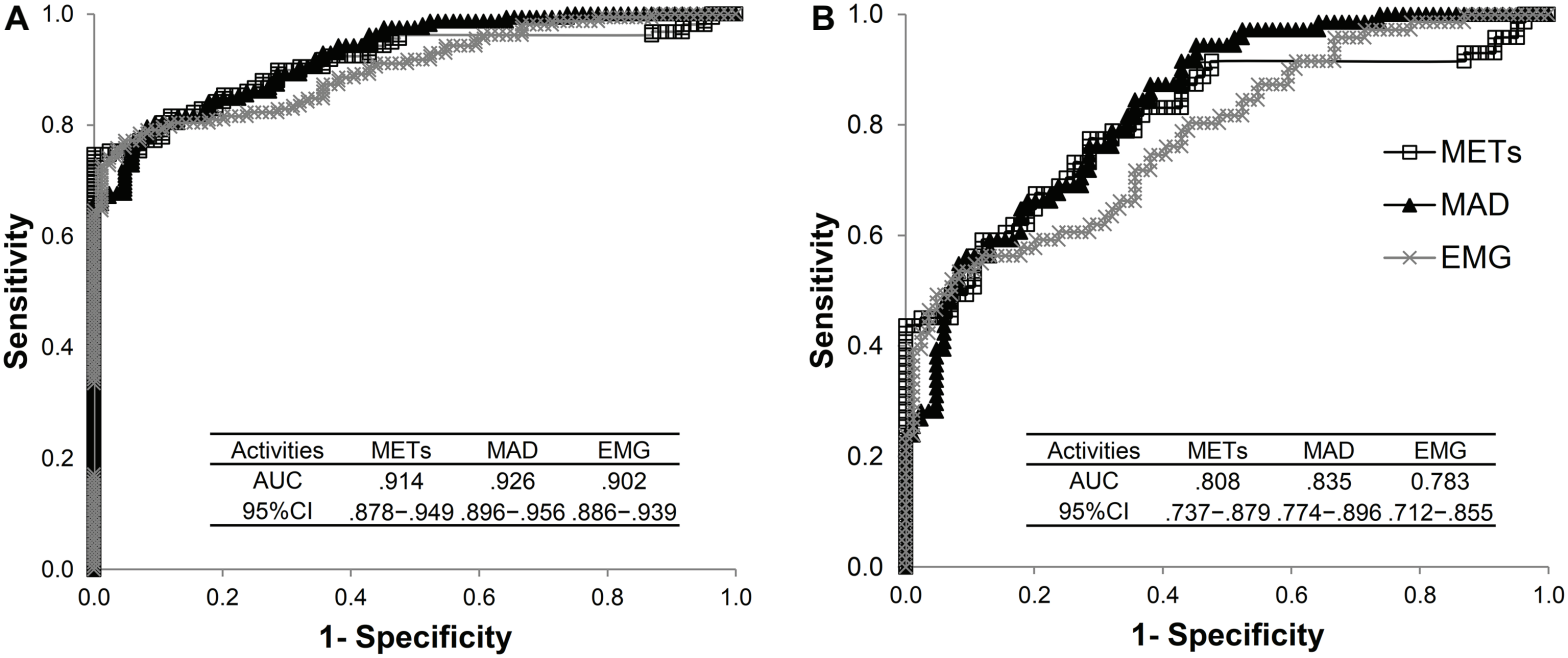
The ability of METs, MAD, and EMG to discriminate sedentary and non-sedentary activities. The area under the curve (AUC) with 95% confidence interval (CI) was determined from the receiver operating characteristic curves. The activities included lying down, sitting quietly, sitting while playing mobile game, standing quietly, standing while playing mobile game, walking on a treadmill at 4 and 6 km/h, and self-paced walking **(A)**. The activities included lying down, sitting quietly, sitting while playing mobile game, standing quietly, and standing while playing mobile game **(B)**.

The AUCs with their 95% CI for METs, MAD, and EMG for classifying sedentary activities are shown in Figure 2A. The optimal cut-offs for discriminating sedentary and non-sedentary activities were 1.3 for measured METs (sensitivity = 81.6%, specificity = 88.1%), 0.0033 g for MAD (sensitivity = 80.4%, specificity = 90.5%), and 11.9% EMG (sensitivity = 79.1%, specificity = 91.7%).

The corresponding AUC with their 95% CI when walking-related activities were excluded from the analyses is presented in Figure 2B. The optimal cut-offs to discriminate lying down or sitting from standing were 1.2 for measured METs (sensitivity = 77.5%, specificity = 71.4%), 0.0025 g for MAD (sensitivity = 76.1%, specificity = 71.4%), and 9.5% for EMG (sensitivity = 56.3%, specificity = 88.1%).

### Individual Correlations of Mean Amplitude Deviation and Electromyography to Metabolic Equivalent of Tasks

**Figure 3:**
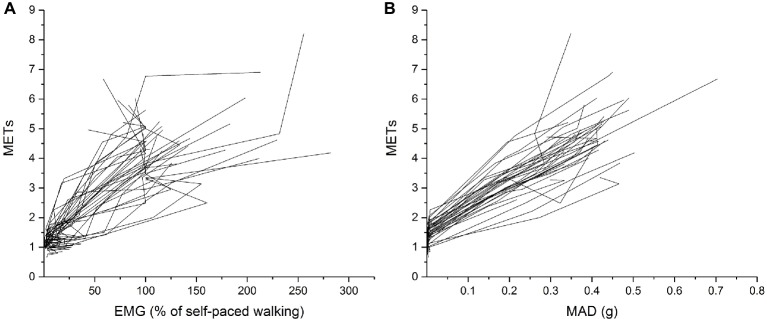
Individual MAD **(A)** and EMG **(B)** plotted against METs during all activities. The activities included lying down, sitting quietly, sitting while playing mobile game, standing quietly, standing while playing mobile game, walking on a treadmill at 4 and 6 km/h, and self-paced walking.

Within individuals, a strong positive mean correlation was found between METs and MAD (*r* = 0.982) and between METs and EMG (*r* = 0.950; Figure 3). In all participants, the MAD or EMG was increased with increasing METs for all activities (all *p* < 0.05).

## Discussion

We found that energy expenditure, movement sensing, and muscle activity were able to discriminate sedentary from non-sedentary activities with acceptable sensitivity and specificity. However, their ability to discriminate sedentary activity from standing was poorer, and the probability for false positive and false negative classification increased. Nevertheless, somewhat reasonable classification performance was still maintained, and relatively similar cut-off was found as compared to when all non-sedentary activities were considered.

In line with previous studies (Evenson et al., 2008; Trost et al., 2011), we found that movement sensing had acceptable sensitivity and specificity to differentiate sedentary activities from non-sedentary activities. However, comparison of physical activity and sedentary outcomes between different studies is not straightforward because different devices utilize different metrics and algorithms (Migueles et al., 2017). Our study is one of the first providing cut-off for sedentary activity using MAD in children less than 13 years of age. MAD may overcome many problems related to arbitrary counts reported in previous studies. MAD is based on the raw acceleration data and allows a direct comparison between different accelerometer brands (Vähä-Ypyä et al., 2015b). However, to the best of our knowledge, only two studies have investigated the cut-off values of MAD to separate sedentary activities from non-sedentary activities in adolescents and adults (Aittasalo et al., 2015; Vähä-Ypyä et al., 2015b). Those studies included standing-related activities into sedentary activities (Aittasalo et al., 2015; Vähä-Ypyä et al., 2015b). This has obscured our understanding on thresholds of sedentary activities in children because standing should be considered separate element from sedentary behavior as it has been found to exhibit higher energy expenditure and muscle activity than sitting (Mansoubi et al., 2015; Gao et al., 2017). Previous study reported 0.0167 g as an optimal cut-off to differentiate between sedentary and non-sedentary behaviors (Vähä-Ypyä et al., 2015b), which is larger than that was observed at 0.0033 g in the present study. Further, this value slightly decreased to 0.0025 g when we considered only non-sedentary activities without walking-related activities. While our results suggest a lower cut-off for sedentary activities in children than in adolescents and adults, it is unclear to what extent this reflects actual differences between children and adults, e.g., the wider pelvis of adults and associated higher accelerations caused by any rotational pelvic movement, or if this is caused by the differences between measurement protocols.

Complexity and large inter-individual variation of sedentary behavior in children with often short intermittent bouts of different sedentary activities at different activity and energy expenditure levels interspersed with non-sedentary activities make the assessment of sedentary activities using movement sensing challenging (Mansoubi et al., 2015). We found that METs, MAD, and EMG were higher during standing than sitting or lying down. Furthermore, METs, MAD, and EMG were able to separate sedentary activities from non-sedentary activities with good sensitivity and specificity, but the ability to separate lying down and sitting from standing was much weaker. These results suggest that different methods can be used to differentiate sedentary activities from non-sedentary activities including movement with relatively good accuracy, but the discrimination between lying down or sitting and standing is much less precise. Furthermore, we found that MAD was increased with increasing walking velocity, while for lying down, sitting, and standing-related activities, MAD values remained consistently low. Importantly, we found more variation between children in METs and EMG than in MAD in lying down and sitting- and standing-related activities. This observation suggest that one fixed cut-off based on movement sensing may not completely capture sedentary behavior in children, and therefore, studies investigating whether individualized cut-offs for sedentary behavior based on posture, energy expenditure, and accelerometry improve the classification accuracy are warranted. To this end, we have also presented MAD values as percentage of self-paced walking (Figure 1), and this approach should be further investigated whether it could take into account individual’s functional capacity and therefore better reflect the individual’s energy requirement.

Because of a strong positive correlation of MAD and EMG with METs, our results suggest that MAD and EMG can be used as surrogates of energy expenditure in activities with varying intensity mimicking activities found in free-living conditions. When we evaluated during sitting or standing quietly vs. while playing mobile game, EMG, but not MAD, was able to discriminate quiet sitting or standing from playing in a sitting or standing position. On the other hand, both EMG and MAD were similarly sensitive and specific to discriminate sedentary from non-sedentary behavior with cut-off values of 11.9% of EMG during self-paced walking and 0.0033 g, respectively. It is important to notice that MAD is an absolute measure, while EMG threshold is related to individual’s effort (as percentage during self-paced walking), suggesting the EMG can supplement accelerometry recordings providing individualized approach to the threshold values. Furthermore, in the present study, MAD values were obtained from hip-worn accelerometry, whereas thigh-worn devices, particularly when utilizing the device orientation to indicate upright/horizontal, may better distinguish postures like sitting and standing compared to hip-worn devices (Edwardson et al., 2016).

Muscle activity has been hypothesized to be a key physiological stimulus in preventing the detrimental effects associated with sedentary behavior, sitting in particular (Hamilton et al., 2007). Accordingly, standing, which requires activation of the anti-gravity muscles, should be considered a non-sedentary behavior (Mansoubi et al., 2015; Gao et al., 2017) and be differentiated from sitting. For example, some of the cardio-metabolic benefits of replacing sitting with standing may be accounted for by (1) a higher muscle activation during standing vs. sitting; (2) a higher muscle activation in overweight vs. normal weight people (the overweight get larger benefits from these trials); and (3) inter-individual variability in muscle activation during sitting and standing (Pesola et al., 2016). Anecdotally, and as seen in the present study, it is not entirely trivial to differentiate sitting from standing in free-living conditions using contemporary wearable devices and analysis methods, but being able to differentiate between the two is a key requirement in order to develop a nuanced understanding of the consequences of sedentary and non-sedentary behaviors. Thus, differentiating between sedentary and non-sedentary behaviors may yield in-depth information for future interventions targeting sedentary behavior. Importantly, a similar volume of total energy expenditure can be accumulated with wildly varying combinations of sedentary and non-sedentary behaviors, and the effects of specific combinations on health outcomes are, thus far, poorly understood.

The strengths of the present study include the use of three different methods to assess sedentary threshold and their ability to discriminate sedentary activities from non-sedentary activities with and without standing-related activities. However, we did not evaluate the usefulness of wrist-worn accelerometers to assess sedentary threshold, and therefore, the thresholds provided in the present study are not translatable for studies using only wrist-worn accelerometry. We also directly measured REE, which allowed us to use child-specific MET values. Because previous studies have not collected data on individual REE, their analyses are based on adult MET value (Aittasalo et al., 2015; Saint-Maurice et al., 2016). However, our study sample was relatively lean and included children aged 7–11 years, which may hinder the generalizability of our results to overweight or obese children and to adolescents. Furthermore, because our sample pooled children aged 7–11 years, we cannot exclude the possibility that the sedentary threshold varies between different age-groups. Younger children have been found to have higher REE than older children (Harrell et al., 2005). We also used MET values normalized for body mass, which may have influenced our results because body mass includes fat mass that has smaller effect on energy expenditure than muscle mass (Tompuri, 2015).

## Conclusion

We found that measured METs, open-source accelerometry analysis, and EMG can be used to differentiate sitting and standing, and sedentary behaviors from physical activities with appropriate sensitivity and specificity. When validated thresholds are used, we can gain understanding of the specific constructs of sedentary behavior, which link it to several health and development outcomes already at childhood.

## Data Availability

The datasets generated for this study are available on request to the corresponding author.

## Ethics Statement

The Ethics Committee of the University of Jyväskylä.

## Author Contributions

YG, EH, AS, and TF, conceived and designed the experiments. YG and AV performed the experiments. YG, AV, and TR analyzed the data. YG wrote the first draft of the manuscript. YG, EH, AL, AP, TR, and TF revised it critically for important content. All authors approved the final draft of the manuscript submitted for review and publication.

### Conflict of Interest Statement

The authors declare that the research was conducted in the absence of any commercial or financial relationships that could be construed as a potential conflict of interest.
